# Geographically closed, yet so different: Contrasting long-term trends at two adjacent sea turtle nesting populations in Taiwan due to different anthropogenic effects

**DOI:** 10.1371/journal.pone.0200063

**Published:** 2018-07-31

**Authors:** I-Jiunn Cheng, Wan-hwa Cheng, Y-T. Chan

**Affiliations:** 1 Institute of Marine Biology, National Taiwan Ocean University, Keelung, Taiwan, ROC; 2 The Masters of Advanced Study in Geographic Information Systems, Arizona State University, Tempe Campus, Arizona, United States of America; National Cheng Kung University, TAIWAN

## Abstract

Marine turtles are endangered megafauna that face both natural disturbances and anthropogenic threats. The islands of Wan-an and Lanyu support two important green turtle nesting populations in Taiwan and are separated by 250 km. Nesting activity was first documented on Wan-an Island in 1992, with 8 nesting females being documented. A further 11 nesting females were first documented on Lanyu Island in 1997. However, by 2015, the Wan-an Island population declined to only 2 nesting females, whereas the Lanyu Island population showed peaks in abundance (up to 24 nesters) every 3–5 years with no long-term decline. Additionally, the recruitment of new nesters to the Wan-an Island population decreased to 15%, whereas recruitment into the Lanyu Island population remained high (66%). The decrease of the nesting population on Wan-an Island might be due to illegal poaching on the high seas along the migratory corridor of the turtles, whereas the stable nesting population on Lanyu Island showed no evidence of such a threat. The two nesting populations use different migratory corridors to their foraging grounds, resulting in different fates of development in population trend.

## Introduction

The primary threats to sea turtle populations include egg harvest, coastal artisanal and commercial fisheries catch and by-catch of turtles, terrestrial and marine habitat alteration, destruction and pollution, pathogens and climate change. Among these threats, fisheries by-catch and coastal development are likely the most important [1–3].

Although Japanese researchers have conducted extensive research on sea turtles; gaps remain in the knowledge for east Asian countries. Political conflicts in the region and limited funding impede research opportunities on sea turtle ecology and the threats that they face, particularly in Taiwan, despite great effort devoted to the nesting ecology of sea turtles at the primary nesting sites e.g. [4–7].

Determining age- and stage-based survivorship patterns is a key to understanding the underlying mechanisms of sea turtle population dynamics and adaptive fitness, which is important for species conservation [8–11]. Increasing survival in the first-year cohort has a positive effect on the overall population trend regardless of the mortality rates during marine life stages. Adult survivorship is also recognized by some studies e.g. [11–13] as the second most important for the survivorship of a population.

Whereas age-class specific survival probabilities and abundance estimates are important to know for determining the status and trends of a sea turtle population, depleted populations are likely to recover if anthropogenic threats can be mitigated [14]. Many studies demonstrate that successful and long-term application of relatively simple and inexpensive conservation practices, such as nest protection and restriction of egg poaching, can help in the recovery of depleted populations [15–20]. This finding suggests that sea turtle populations are sustainable with proper conservation measures that help prevent the Allee effect, which can ultimately lead to extinction due to reduced fitness in declining populations [14].

**Fig 1:**
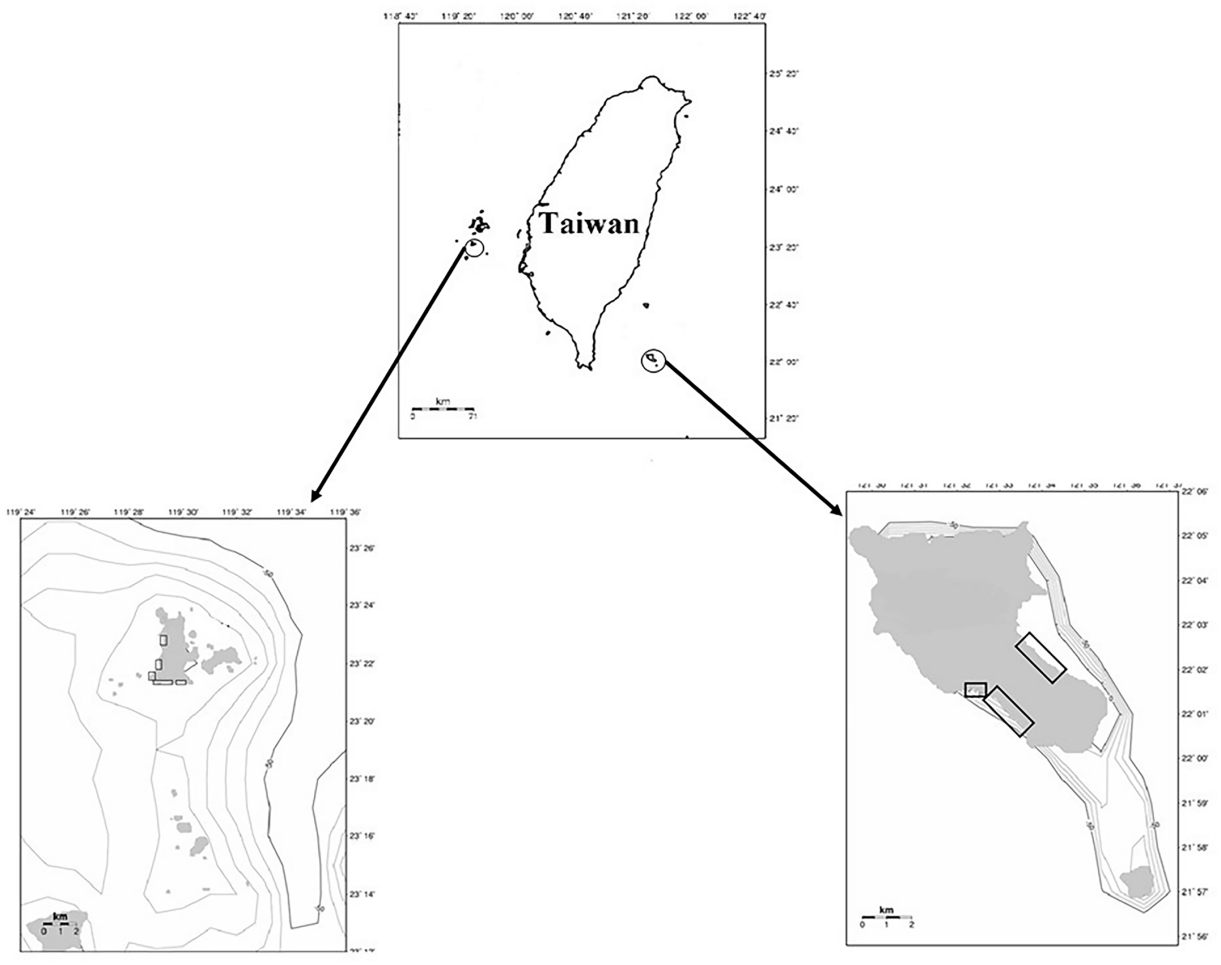
Map of Taiwan with maps of Wan-an and Lanyu islands attached to both sides of Taiwan. The nesting beaches are marked with rectangles.

Taiwan has three primary sea turtle rookeries (Fig 1): Wan-an Island in the Penghu Archipelago, Lanyu Island in Taitung County and Liuchiu Island in Pengtung County. Knowledge is lacking on the population trends for green turtles (*Chelonia mydas*) at both the Wan-an Island and Lanyu Island rookeries over the past 18 years. The objectives of the present study were to determine the long-term trends and the factors affecting these two different populations.

## Materials and methods

Ethics Statement—All applicable international, national, and/or institutional guidelines for the care and use of animals were followed. The study was possible under the Affidavit of Approval of Animal Use Protocol College of Life Sciences, NTOU (No. 105054) and the Forestry Bureau, Council of Agriculture, Republic of China (Agri Forest 1061700491). For Wan-an Island, the permission to work on the island was issued by Penghu County, whereas no specific permission was required on Lanyu Island. However, all the works on sea turtles were possible with permission issued by the Forestry Bureau, Council of Agriculture. We did not sacrifice any sea turtles for the experiments, and all sampling procedures and experimental manipulations were specifically approved as part of obtaining the field permit. Full details of collection and sampling methods are listed in the Materials and Methods sections.

### Study sites

#### Wan-an Island (Fig 1)

Wan-an Island (23°22’ N, 119°30’ E) is located in the southern Penghu Archipelago, approximately 18 miles from the main Penghu Island. The island is the fourth largest island in the archipelago and is approximately 4 km long and 1.8 km wide, with an area of approximately 7.17 km^2^. The average annual air temperature is 22°C and the average annual precipitation is 1117 mm, which falls primarily in the monsoon and typhoon seasons.

The island has 11 relatively flat sandy beaches, all composed of quartz porphyries, coral and shell debris. These beaches range from 67 to 800 m in length, ranging from 20 to 100 m in width, and are separated by rocky outcrops. Turtles emerge on 9 of the beaches, all of which are located on the south and west sides of the island. The total length of beach that is used by the green turtles for nesting is approximately 4 km [21,22].

#### Lanyu Island (Fig 1)

**L**anyu Island (22°00’ to 08’ N, 121°50’ to 60’ E) is in the Pacific Ocean southwest of Taitung, Taiwan, and has an area of approximately 45.74 km^2^. The island has typical tropical rainforest weather, with average annual sunshine of 1490 hours, annual average air temperature of 22.4°C, annual precipitation exceeding 3077 mm and annual relative humidity of approximately 90% [23]. The sand on the beaches is quartz porphyrite that is occasionally inter-layered with muddy sediment.

Although 6 sandy beaches are on Lanyu Island, a preliminary survey showed nesting activity on only three of the beaches. The areas of these three beaches were approximately 5522, 17,000 and 15,766 m^2^. The length of each beach ranged from 400 to 900 meters [24].

### Methods

#### Environmental data

Sand grain characteristics.

Sand samples were taken adjacent to the nesting sites and were stored dry in double-sealed plastic bags. The graphic mean (Mz; in mm) and inclusive graphic standard deviation (σ_1_) of the beach sand were determined according to [25]:

$$M_{z}(\phi )=(\phi_{16}+\phi_{50}+\phi_{84})/3$$

$$\sigma_{1}=\;(\phi_{95}-\phi_{5})/6.6+(\phi_{84}-\phi_{16})/4.4$$

where *ϕ*_16_, *ϕ*_50_, *ϕ*_84_, *ϕ*_95_, and *ϕ*_5_ denote the proportion by weight of total sand at 16, 50, 84, 95 and 5%, respectively, of the total phi (*ϕ*) value. The sand samples were collected from 1997 to 2015 from both islands.

#### Climate data

The meteorological data collected for this study included daily air temperature and precipitation on both Wan-an Island and Lanyu Island from 1997 until 2006. The data were purchased from the Central Weather Bureau of the (Republic of China) ROC (Central Weather Bureau, 1997–2015). Meteorological data on Wan-an Island were obtained from weather stations on two nearby islands. Meteorological data on Lanyu Island were obtained from the weather station on Lanyu Island.

#### Biological data

The nesting ecology of green turtles was studied on both islands from the last week of June until the middle or end of September between 1997 and 2006. The nesting season on Wan-an Island lasts from mid- to late June until early October. The nesting season on Lanyu Island lasts the entire year, but nesting activity occurs primarily from late June until the end of September. A survey program was conducted that covered the primary nesting activities of the year. Both Wan-an and Lanyu islands are small, and the turtles nested randomly on all the beaches. Thus, we considered each island as a single nesting site.

On each island, three categories of parameters were collected each year: nesting behavior of the female turtles, incubation physiology and hatchling morphology. The sampling protocols used are reported in [4,5]. Briefly, we measured the straight and curved carapace lengths from the midpoint of the nuchal scute to the posterior-most tip of the carapace (SCL and CCL, [26]) of each nesting female and tagged the trailing edge of the front and rear flippers using numbered Inconel tags (National Band and Tag Co., Style 1001–681). For the nesting behavior surveys, we recorded the number of nesters, nesting dates, number of emergences, clutch size (number of eggs), and clutch frequency (number of nests per turtle per season). From these data, we calculated the inter-annual remigration intervals (years), inter-nesting interval (days) and nesting success (%). The inter-annual remigration interval was determined by calculating the number of tagged turtles that were observed nesting in more than one season. Turtles that had not been tagged or that did not have any evidence of prior tagging (e.g., tag scars) were classified as ‘new’ turtles, whereas turtles that had been tagged on the same beach or those bearing tag scars were classified as previously recorded nesters [27]. The remigration interval is defined as the number of years between two nesting seasons for the same female turtle. The inter-nesting interval is defined as the number of days between nesting events within the same season [28]. Nesting success was defined as the percentage of emergences that resulted in egg deposition.

To examine incubation physiology, we recorded the following data for each nest: the total number of eggs, nest depth, incubation period (days), number of eggs not hatched, and egg size (cm) and weight (g) for a random sub-sample of 30 eggs per clutch.

The nest depth was measured from the surface of the beach to the bottom of the nest in centimeters (cm). The number of fertilized eggs was determined after the nest hatched by counting the empty eggshells and adding the number of dead fetuses in unhatched eggs [29]. From these data, we calculated hatching success (%), reproductive output (eggs) and clutch survival rate (%). Hatching success was calculated as the percentage of live hatchlings from the total clutch. The post-hatch mortality rate (%) was calculated as the percentage of dead hatchlings from the total number of hatchlings. The emergence rate (%) was calculated as the percentage of live hatchlings that emerged from the nest. The reproductive output is typically expressed as the total egg production of all females on the nesting beach during each season. However, because of logistical difficulties (e.g., typhoons or high waves), recording the size of every clutch was impossible. Therefore, the reproductive output was estimated by multiplying the total number of nesting females by the mean clutch size and the mean clutch frequency [30,31]. Clutch survival was calculated as the product of hatching success and emergence success. Emergence success was defined as the percentage of hatchlings emerging onto the beach from the nest [5].

Animal biologging data are used to assess mortality rates of both terrestrial and marine animals [32,33]. The signal that infer the death of animal, especially the mairne animal, is stop transmitting during the early phase of migration in the open space or ocean which the shedding or malfunction of the tag is highly impossible. In this study, satellite telemetry was used to follow post-nesting females that departed from Wan-an and Lanyu islands. Animals were tracked with satellite telemetry from both islands. Seven nesting green turtles, three from Wan-an Island in 2010, 2011 and 2013 and four from Lanyu Island in 2010, 2012, 2012 and 2014, were equipped with Satellite-Relayed Data Loggers (SRDL 9000X; Sea Marine Research Unit, SMRU, St Andrews, UK, http://www.smru.st-and.ac.uk/; unit mass = 660 g). One rehabilitated turtle was also equipped with the same satellite tag. The basic morphological information and deployment date are showed in table in S1 Table. SRDL units were glued on the highest scale of the carapace using 2-component fast set epoxy (Powerfast), with the antenna pointing perpendicular to the sea surface to improve communication with the satellites upon the turtle surfacing. Tracking and sensor data were then processed and displayed through online platforms such as the Satellite Tracking and Analysis Tool (STAT), originally designed for turtle studies [34]. Argos locations of any accuracy were used except those implying an apparent speed above 1.5 m s^−1^ (i.e., 5 km h^−1^), as travel rates above this threshold are considered biologically unlikely [35,36].

Student’s t-test was used to compare the differences in parameters between Wan-an and Lanyu islands [37]. The percentage values were divided by 100, and an arcsine square root transformation was applied before comparisons were performed. Linear regression was used to determine the long-term rates of change in the numbers of nesting females on both Lanyu and Wan-an islands [37].

## Results and discussion

**Fig 2:**
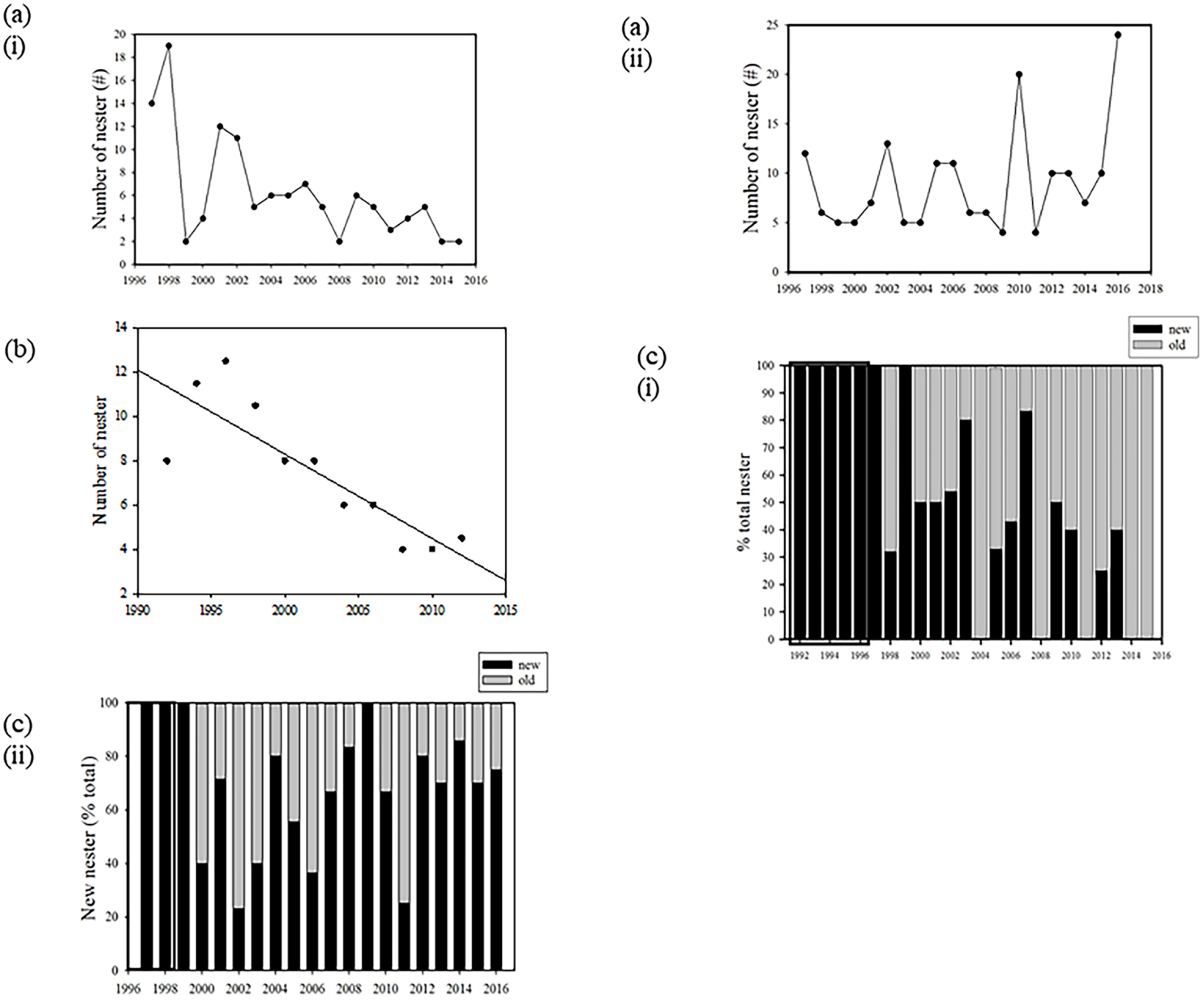
Changes in the nesting population and recruitment rate (proportion of new nesting females) for both the Wan-an Island and Lanyu Island populations. (a) population trend of (i) Wan-an Island rookery (1993–2015) and (ii) Lanyu Island rookery (1997–2015); (b) two-year running mean of Wan-an Island nesting population from 1992 to 2015, with a declining trend observed: number of nesting females = 12.127–0.764(year), n = 11, r = 0.85, *p* < 0.001; (c) proportion of new nesting females in the nesting population across both islands. The dark frame shows the beginning of the field sampling program completed for this study and represents years without any returns of tagged turtles at (i) Wan-an Island and (ii) Lanyu Island.

The nesting population on Wan-an Island increased from 1997 until 1998 and then decreased thereafter to only 2 turtles in 2015 (Fig 2(a)(i), table in S2 Table). However, no significant upward or downward trends were found for the Lanyu Island population during the study period, but abundances peaked every 3 to 5 years (Fig 2(a)(ii), table in S2 Table). These results suggested that the Wan-an Island nesting population was approaching collapse, whereas the Lanyu Island population remained stable. Although the Allee effect is apparently unimportant for sea turtles, other factors such as disease and anthropogenic threats, among others, may cause a population to decline, with the decline very difficult to mitigate and may even result in a trophic cascade [38].

For the long-term study e.g. [39–41], the remigration interval of the sea turtles varied depending on the food availability in the previous year. This value is typically more variable in green turtles than that in other sea turtle species, because differences are driven by the trophic status of the different species. Green turtles are herbivores, feeding primarily on primary producers, such as macroalgae and sea grass. These food sources are influenced primarily by weather patterns; even large-scale climate variations such as ENSO and NAO can affect macroalgae and sea grasses [42]. Furthermore, years that have relatively high nesting frequency are often followed by years with relatively low nesting frequency. This phenomenon is observed because sea turtles must acquire sufficient energy through food to reach the critical condition that is necessary for the initiation of the breeding migration. When a single population has a large proportion of the females nesting in a single year, several years are required before the females can complete another breeding migration; in this study, the remigration interval was typically 5 years. Thus, the nesting populations in the years following a nesting event were likely to be very low.

The variability in inter-annual nesting numbers (i.e., coefficient of variation (CV)) reflects the variation in the foraging conditions and physiological status of the nesters [39]. The variability in the number of nesters was 0.62 for the Wan-an Island population and 0.49 for the Lanyu Island population. These values are within the range found in other green turtle studies; for example, the CV was 0.36 at Patrick Air Force Base in Florida (USA, [43]) and 1.13 for an Israel population [43]; the average CV is 0.76 [39,41,43–48]. However, the inter-annual variability is likely to reflect variation in foraging conditions. Thus, we compared the mean number of nesting females in the first 5 years (1992 to 1996 for the Wan-an Island population; 1997 to 2001 for the Lanyu Island population) to that in the last 5 years (2011 to 2015 for both populations). For the Wan-an Island population, the mean annual nesting number decreased from 7 to 3 turtles per year, whereas the Lanyu Island population increased from 7 to 8 turtles per year. Additionally, to account for inter-annual variation in the nesting number, a 2-year running mean was calculated for both populations. Using the 2-year running mean, a significant decreasing trend for the Wan-an Island population was evident (number of nesters = 12.127–0.764(year), n = 11, r = 0.85, *p* < 0.001; Fig 2(b), table in S3 Table), whereas no such trend was observed for the Lanyu population (number of nesters = 7.417 + 0.15(year), n = 9, r = 0.16, *p* = 0.682). Thus, despite relatively stable conditions in the foraging sites, the Wan-an Island population clearly was collapsing; whereas the Lanyu Island population remained stable. The developmental sites of the two populations were likely in different locations, with those of Wan-an Island possibly affected by environmental degradation or other anthropogenic activities. The two populations might also have different foraging areas or different migratory routes, and this assumption was supported by the animals were tracked with satellite telemetry from both islands (detailed in a later section). The results showed that the end points of post-nesting migrations were different, with the west Pacific for most Lanyu nesters and the East China Sea for most Wan-an nesters.

Factors that lead to changes in population trends include intrinsic factors, such as demographic fluctuations, and extrinsic factors, such as climate change and anthropogenic threats in the marine and terrestrial habitats of the turtles. A ten-year study comparing Wan-an and Lanyu islands suggests that both the environment and the nesting ecology are different [4]. Wan-an Island is hotter and drier than Lanyu Island. Although no differences were found in the size of the nesters between the two populations, females on Wan-an Island deposited larger and heavier eggs, had higher clutch frequencies, dug deeper nests, and their eggs had shorter incubation periods. The inter-nesting intervals were also longer on Wan-an Island. The sediment on the nesting beach of Wan-an Island was finer and more homogenous in distribution than that on the Lanyu Island beaches. Thus, compared with the Lanyu Island population, the Wan-an Island population had greater hatching success but greater post-hatch mortality. Hatchling emergence success was also higher on Wan-an Island (Table 1). However, these differences could not explain the differences in the trends between the two populations. Therefore, other life history traits related to the population trends were examined. The remigration interval of the nesters from Wan-an Island was 4.9±1.9 years (n = 48), and for the nesters from Lanyu Island, the interval was 4.4±1.6 years (n = 37). No significant difference in the remigration interval was found between the islands (*p* = 0.148, df = 84); thus, females returned to their nesting islands at similar intervals.

**Table 1:** Results of comparisons between Lanyu (L) and Wan-an (W) islands from 1998–2014. Significant differences are indicated in bold font.

Parameter	Wan-an	Lanyu	Result	*p*-value	df
Nesting females	7 ± 5 (19)	8 ± 4 (19)	W ~ L	0.129	26
Remigration interval	4.9 ± 1.9 (48)	4.4 ± 1.6 (37)	W ~ L	0.148	84
**Recruitment (%)**	68 ± 26 (19)	46 ± 31 (19)	**L > W**	**0.029**	**38**
Clutch size	107±26(373)	107±27 (372)	W ~ L	0.559	744
**Emergence times**	10 ± 7 (118)	7 ± 5 (145)	**W > L**	**< 0.001**	**262**
**Inter-nesting interval**	14 ± 1 (335)	11 ± 2 (280)	**W > L**	**< 0.001**	**542**
**Clutch frequency**	4 ± 2 (117)	3 ± 2 (152)	**W > L**	**< 0.001**	**268**
**Incubation period**	52.4 ± 3.7 (336)	55.9 ± 4.3 (97)	**L > W**	**< 0.001**	**432**
**Nest depth**	69.2±9.2 (305)	68.2±179(360)	**W > L**	**< 0.001**	**664**
Nesting success	48±26 (118)	46±28 (145)	W ~ L	0.98	262
Hatching success	74 ± 29 (370)	68 ± 34 (306)	W ~ L	0.29	675
Reproductive output (KJ)	329476±223128(19)	281305±165391(19)	W ~ L	0.467	37
Reproductive output (eggs)	2397±1290(19)	2668±1367(19)	W ~ L	0.583	37
Clutch survival rate	64 ± 31 (473)	58 ± 37 (326)	W ~ L	0.149	798

**Fig 3:**
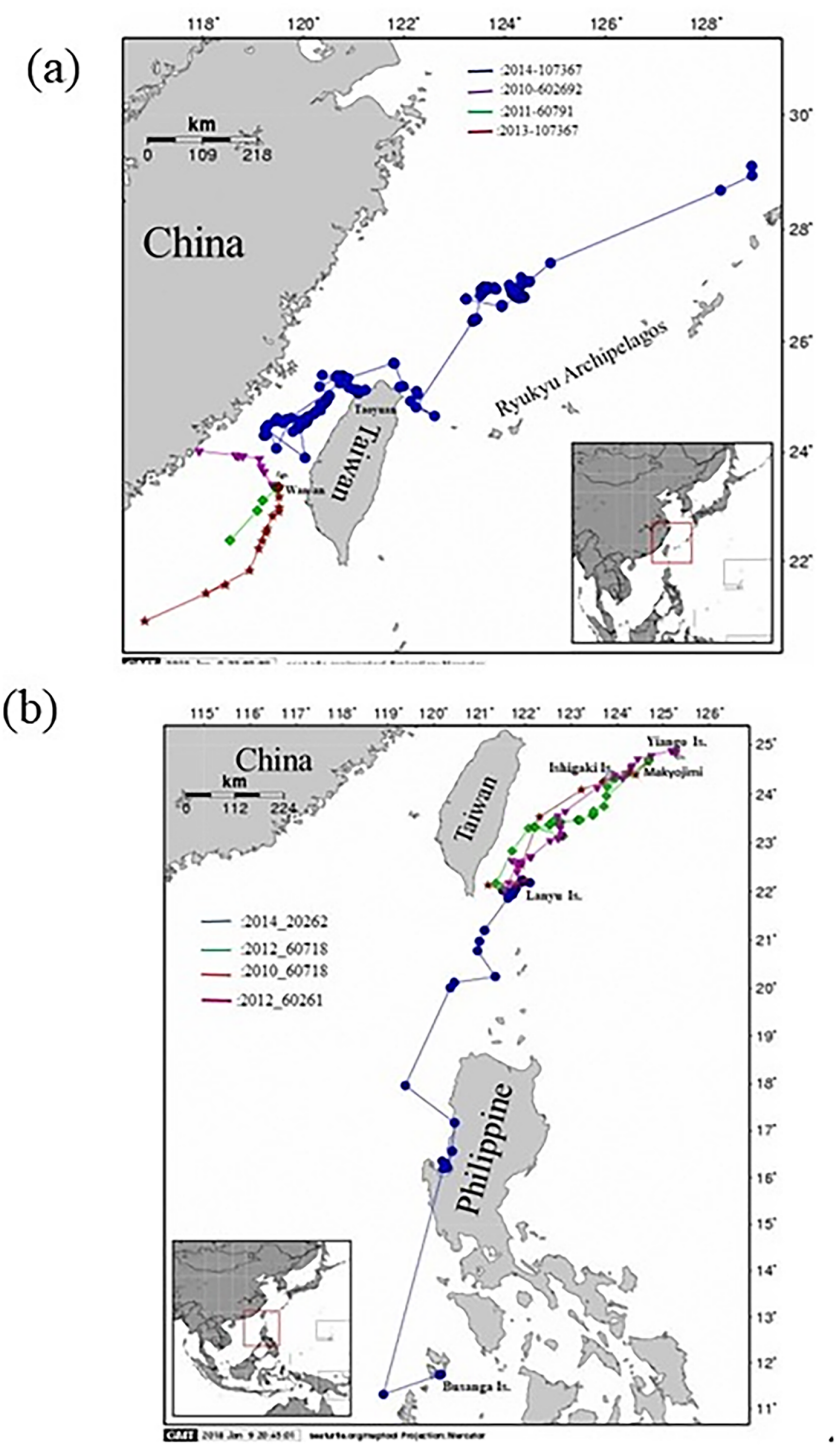
Post-nesting migration of green turtles from (a) Wan-an Island, which included turtle ID 107361 in 2013, turtle ID 602692 in 2010, turtle ID 60791 in 2011, and a rehabilitated turtle with ID 107367 in 2014. (b) from Lanyu island, which included turtle ID 20262 in 2014, turtle ID 60718 in 2012, turtle ID 60718 in 2010, and turtle ID 60261 in 2012.

Among various extrinsic factors, we excluded the influence of climate change. The remigration interval varies between individuals and populations and may be related to individual quality, environmental conditions or climatic cycles. Thus, environmental fluctuations, such as ENSO, can have a substantial influence on the remigration interval. This effect, in turn, influences the reproductive output and eggs per clutch [13,30,42,49–51]. Although the nesting environments are different between the two islands [7], the similar remigration intervals indicated similar responses to global climate change during the study period. Both populations using the same foraging area is another possibility; however, animal were tracked with satellite telemetry showed that two populations had different foraging sites (Fig 3). Additionally, both reproductive output and clutch size were similar between the two populations (Table 1). Furthermore, no long-term changes in air temperature, precipitation or sand characteristics were observed on the nesting beaches at either Wan-an Island or Lanyu Island from 1997 to 2015 (Cheng, unpublished data). We also excluded the possibility of terrestrial anthropogenic threats, because the nesting beaches on Wan-an Island have been designated protected areas since 1995 [52,53]. A saturated monitoring program was conducted that covered the entire nesting period for three years before the designation of the protected area [21]. A management plan, including beach patrols and restricted entrance to the nesting beach at night, has been in place since then to ensure that both nesting females and hatchlings are not subject to human interference. Ten islands are near Wan-an Island; however, fewer than 4 nests were recorded on only one of those islands each year. Thus, the decline of the nesting population on Wan-an Island could not be attributed to individual nesters using the other beaches for nesting.

By contrast, the nesting beaches on Lanyu Island have not been designated protected areas to date. Several anthropogenic threats occur, including sand mining, turtle tourism, light pollution by houses and bars near the nesting beaches, and occasional turtle poaching by sport fishermen [5]. However, the trend in the sea turtle population on this island was apparently unaffected by these threats. The aboriginal people living on Lanyu Island possibly did not hunt sea turtles or poach the nesting females, eggs or their hatchlings, because they believed that the sea turtles nested near their graveyards, which were guarded by spirits or evil ghosts. Thus, the nesting females are forced to select less suitable nesting sites under the existing anthropogenic threats, such as beaches under street lights. Moreover, the simple and relatively inexpensive strategy of nest protection and restricting egg poaching failed to recover the Wan-an Island population, although the strategy has helped other populations [14,17–20].

The difference in trends of the nesting populations between the two islands might be due to differences in their exposure to anthropogenic threats at sea. Studies of population dynamics suggest that populations increase or decrease depending on the proportion of new recruits arriving to nest each year [54]. An increase in recruitment results in an increasing population trend, whereas a decrease in recruitment results in a declining population trend. Unfortunately, the rate of recruitment is difficult to determine in long-lived species, such as sea turtles, particularly because sea turtles exhibit highly variable nesting numbers and recruitment rates. The trends in population dynamics and recruitment cannot be determined with only a few years of data [16,19,55]. Thus, a longer-term study of sea turtle ecology is required to determine the possible causes of changes in the nesting populations at Wan-an and Lanyu islands.

We calculated the changes in yearly proportions of recruitment for both islands. Average recruitment on Wan-an Island was 46% from 1997 to 2015 and decreased to 15% when only the 5 most recent years were considered. Average recruitment was 48% during the same period on Lanyu Island and remained constant (66%) in the 5 most recent years (Fig 2(c), table in S4 Table). Recruitment was higher on Lanyu Island than that on Wan-an Island (*p* = 0.029; online supplement Table). This finding indicated that the juvenile populations were differentially affected on the two islands, leading to different levels of recruitment. The two populations likely have different developmental sites, with those of Wan-an Island affected by some environmental degradation or anthropogenic activities. Based on the results of satellite studies (detailed in a later section), the tags from Wan-an nesters stopped transmitting in the Taiwan Straits, whereas the tags from Lanyu nesters continued transmitting until batteries drained in the foraging sites. Therefore, the results of this study highlight the necessity to locate the developmental sites of these two populations.

The decline in recruitment observed in this study might be the primary reason for the collapse of the nesting population on Wan-an Island because such a decline lowers the reproductive output and reduces the population over time. Many studies e.g., [19,56–60] suggest that the direct harvest of sea turtles, particularly subadults and nesting females, can greatly affect the sea turtle population. Similar to other K-selected species, long-term stability of sea turtle populations depends on the longevity of adult individuals, which requires a relatively safe environment. Thus, removing adult individuals from the population can easily cause a population to collapse. The migratory nature of sea turtles may subject them to various threats from countries adjacent to their migration routes [61].

Based on the results of satellite studies that followed the post-nesting females departed from Wan-an Island, data transmission ceased abruptly in the Taiwan Straits during the early phases of migration in 2006, 2010 and 2011, and transmission ranged from 5 to 21 days after the start of the post-nesting migration. Even the transmissions from a rehabilitated and released turtle ceased on the continental shelf between the Ryukyu Archipelagos and Kyushu, Japan (Fig 3a) 107 days after release. However, the satellite telemetry data from females that nested on Lanyu Island in 2010, 2012 and 2014 showed that those individuals completed their post-nesting migration and located their foraging sites in the nearshore waters of the southern Ryukyu Archipelago (adjacent to Iriomote, Ishigaki and Miyako-jima) and the southern Philippines (e.g., south of Busuanga Island). Those tags ceased transmitting data after 2 to 10 months, once their batteries were drained (Fig 3b). Satellite telemetry data allow researchers to locate the primary foraging sites of tagged turtles, although some turtles can use multiple sites within a year (e.g. [62,63]). In this study, we accurately located the foraging sites of the nesters from Lanyu Island, but not the sites of those from Wan-an Island. Thus, the decline in the Wan-an Island nesting population might be related to fishing mortalities on the high sea when the nesters are migrating to their foraging sites. By contrast, nesters from Lanyu Island use the Kuroshio Current to reach their foraging sites after the nesting season. [18] suggested that fishing activities could lead to a population decline, regardless of the conservation efforts on the beach.

The continental shelves east of the Chinese mainland are fished constantly from Chinese ports (e.g., [33]). Results of satellite telemetry studies (Fig 3 and table in S1 Table) suggested that fishermen from China might poach the nesting females from Wan-an Island, whereas those from Lanyu Island were under no such threat. Although China is a signatory nation of the Convention on International Trade in Endangered Species (CITES) and other protective treaties, illegal poaching of sea turtles on the high seas and the trade of shell products (*bekko*) remain very active, with the demand for such products increasing e.g. [33,64].

The decline in nesting populations on Wan-an Island might be due to illegal poaching on the high seas, whereas the stable nesting population on Lanyu Island showed no evidence of such a threat. This result strongly suggested that anthropogenic factors could effectively decrease sea turtle recruitment and cause a population to collapse, particularly for a small population such as that nesting on Wan-an Island. The effective population size of this rookery could drop to a level that is sufficiently low to result in an Allee effect. It is hypothesized that the low population size would be unable to cope with demographic and environmental variations, thereby driving the population to extinction [55].

This study demonstrated that an anthropogenic stress resulted in different population trends at two nearby rookeries over a relatively long time period. Both Wan-an and Lanyu islands have small green turtle populations. The effects of other sources of population variation were minor compared with the population recruitment rate between these two populations. The turtle population nesting at the Wan-an Island rookery is now very much at risk. By contrast, the population on Lanyu Island is stable, which suggested that if recruitment is similar in the future, the population is likely to survive. One additional issue deserves attention: [63] found that male turtles breed more frequently than female turtles, and therefore, a more balanced operational sex ratio is reached in the population. A more recent study demonstrated that the functional operational sex ratio (i.e., receptive females and males at any one time) is consistent through the breeding period, but the total ratio (i.e., total number of males and females breeding in the season) is not. Thus, whereas males are breeding more frequently; the overall relative numbers might not be equal in many populations [65]. Although we did not assess the contribution of male turtles to the nesting populations in this study, the contribution of males remains an important objective for future research.

## Conservation implications

The satellite telemetry data from both islands enabled us to identify the foraging sites for the Lanyu Island population. However, the foraging sites for the Wan-an Island population were not identified, except for some possible sites that included Donsha Atoll in the northern South China Sea, Hong Kong, or the Ryukyu Archipelagos [66]. An animal has arrived at a foraging site when results of satellite studies show that the animal stays in one area for more than two weeks. Several recent studies using the fastlog-GPS to determine quantatively whether a turtle is foraging or at an intermediary point during the migration [62, 63, 67] This will provide a useful tool to identify the foraging and development sites. The conservation measures can then be implemented, which should include a bi-lateral sea turtle conservation memorandum of understanding between Taiwan and China, effective enforcement by the Fisheries Administration of China to stop sea turtle fishing on its continental shelves, and an active sea turtle conservation awareness campaign in China. Otherwise, the population at Wan-an Island is likely to go extinct. A regional and international sea turtle management treaty (e.g., the Inter-American Convention) that includes China, Japan and the Philippines is also desperately needed to reduce the pressure on sea turtles at a regional scale, including China, Japan and the Philippines [68–71].

## Supporting information

S1 Table Deployment site, year, date, size (straight carapace length, SCL), traveled days, residual period in the residual site and figures of the satellite tagged turtles from both Wan-an and Lanyu islands from 2010 to 2014. (DOC)

S2 Table Yearly change in nesting population of Wan-an and Lanyu populations from 1997 till 2015. (DOC)

S3 Table Two years mean of the nesting population from (a) Wan-an population, (b) Lanyu population. (DOC)

S4 Table Ratio of new recruit: Old turtle of the nesting population of (a) Wan-an population from 1992 till 2015, (b) Lanyu population from 1997 till 2016. (DOC)
