# Supplementary material for: Geographically closed, yet so different: Contrasting long-term trends at two adjacent sea turtle nesting populations in Taiwan due to different anthropogenic effects
Source: PLoS One. 2018 Jul 31;13(7):e0200063. doi: 10.1371/journal.pone.0200063 (PMC6067716; doi:10.1371/journal.pone.0200063)
Supplement: S1 Table — (DOC) [file pone.0200063.s001.doc]

| S1 Table. Deployment site, year, date, size (straight carapace length, SCL), traveled days, residual period in the residual site and figures of the satellite tagged turtles from both Wan-an and Lanyu islands from 2010 to 2014. | | | | | | | |
| --- | --- | --- | --- | --- | --- | --- | --- |
| Site | Year | Tag number | Date of deployment | SCL (mm) | Traveled days | Residual period (d) | Figure |
| Wan-an | 2010 | 602692 | 23-Jul | 93 | 5 | 0 | 3 |
| Wan-an | 2011 | 60791 | 9-Aug | 102 | 13 | 0 | 3 |
| Wan-an | 2013 | 107367 | 28-Jul | 106 | 21 | 0 | 3 |
| Wan-an | 2014 | 107367 | 1-Apr | 99 | 107 | 0 | 3 |
| Lanyu | 2010 | 60718 | 15-Aug | 111 | 10 | 315 | 4 |
| Lanyu | 2012 | 60718 | 18-Jul | 115 | 9 | 273 | 4 |
| Lanyu | 2012 | 60261 | 30-Jul | 100 | 44 | 59 | 4 |
| Lanyu | 2014 | 60262 | 16-Jul | 98 | 16 | 145 | 4 |
