# Supplementary material for: Geographically closed, yet so different: Contrasting long-term trends at two adjacent sea turtle nesting populations in Taiwan due to different anthropogenic effects
Source: PLoS One. 2018 Jul 31;13(7):e0200063. doi: 10.1371/journal.pone.0200063 (PMC6067716; doi:10.1371/journal.pone.0200063)
Supplement: S2 Table — (DOC) [file pone.0200063.s002.doc]

S2 Table. Yearly change in nesting population of Wan-an and Lanyu populations from 1997 till 2015.

| year | Wan-an population | Lanyu population |
| --- | --- | --- |
| 1997 | 14 | 12 |
| 1998 | 19 | 6 |
| 1999 | 2 | 5 |
| 2000 | 4 | 5 |
| 2001 | 12 | 7 |
| 2002 | 11 | 13 |
| 2003 | 5 | 5 |
| 2004 | 6 | 5 |
| 2005 | 6 | 11 |
| 2006 | 7 | 11 |
| 2007 | 5 | 6 |
| 2008 | 2 | 6 |
| 2009 | 6 | 4 |
| 2010 | 5 | 20 |
| 2011 | 3 | 4 |
| 2012 | 4 | 10 |
| 2013 | 5 | 10 |
| 2014 | 2 | 7 |
| 2015 | 2 | 10 |
