# Supplementary material for: Geographically closed, yet so different: Contrasting long-term trends at two adjacent sea turtle nesting populations in Taiwan due to different anthropogenic effects
Source: PLoS One. 2018 Jul 31;13(7):e0200063. doi: 10.1371/journal.pone.0200063 (PMC6067716; doi:10.1371/journal.pone.0200063)
Supplement: S3 Table — (DOC) [file pone.0200063.s003.doc]

S3 Table. Two years mean of the nesting population from (a) Wan-an population, (b) Lanyu population.

(a)

| year | Wan-an population |
| --- | --- |
| 1992 | 8.0 |
| 1994 | 11.5 |
| 1996 | 12.5 |
| 1998 | 10.5 |
| 2000 | 8.0 |
| 2002 | 8.0 |
| 2004 | 6.0 |
| 2006 | 6.0 |
| 2008 | 4.0 |
| 2010 | 4.0 |
| 2012 | 4.5 |

(b)

| year | Lanyu population |
| --- | --- |
| 1997 | 9 |
| 1999 | 5 |
| 2001 | 10 |
| 2003 | 5 |
| 2005 | 11 |
| 2007 | 6 |
| 2009 | 12 |
| 2011 | 7 |
| 2013 | 8.5 |
