# Supplementary material for: Geographically closed, yet so different: Contrasting long-term trends at two adjacent sea turtle nesting populations in Taiwan due to different anthropogenic effects
Source: PLoS One. 2018 Jul 31;13(7):e0200063. doi: 10.1371/journal.pone.0200063 (PMC6067716; doi:10.1371/journal.pone.0200063)
Supplement: S4 Table — (DOC) [file pone.0200063.s004.doc]

S4 Table. Ratio of new recruit: old turtle of the nesting population of (a) Wan-an population from 1992 till 2015, (b) Lanyu population from 1997 till 2016

| (a) | | | |  |
| --- | --- | --- | --- | --- |
| year | new recruit | old turtle | |  |
| 1992 | 100 | 0 | |  |
| 1993 | 100 | 0 | |  |
| 1994 | 100 | 0 | |  |
| 1995 | 100 | 0 | |  |
| 1996 | 100 | 0 | |  |
| 1997 | 100 | 0 | |  |
| 1998 | 32 | 68 | |  |
| 1999 | 100 | 0 | |  |
| 2000 | 50 | 50 | |  |
| 2001 | 50 | 50 | |  |
| 2002 | 54 | 46 | |  |
| 2003 | 80 | 20 | |  |
| 2004 | 0 | 100 | |  |
| 2005 | 33 | 66 | |  |
| 2006 | 43 | 57 | |  |
| 2007 | 83 | 17 | |  |
| 2008 | 0 | 100 | |  |
| 2009 | 50 | 50 | |  |
| 2010 | 40 | 60 | |  |
| 2011 | 0 | 100 | |  |
| 2012 | 25 | 75 | |  |
| 2013 | 40 | 60 | |  |
| 2014 | 0 | 100 | |  |
| 2015 | 0 | 100 | |  |
| (b) | | | | |
| year | new recruit | | old turtle | |
| 1997 | 100 | | 0 | |
| 1998 | 100 | | 0 | |
| 1999 | 100 | | 0 | |
| 2000 | 40 | | 60 | |
| 2001 | 71 | | 29 | |
| 2002 | 23 | | 77 | |
| 2003 | 40 | | 60 | |
| 2004 | 80 | | 20 | |
| 2005 | 56 | | 44 | |
| 2006 | 36 | | 64 | |
| 2007 | 67 | | 33 | |
| 2008 | 83 | | 17 | |
| 2009 | 100 | | 0 | |
| 2010 | 67 | | 33 | |
| 2011 | 25 | | 75 | |
| 2012 | 80 | | 20 | |
| 2013 | 70 | | 30 | |
| 2014 | 86 | | 14 | |
| 2015 | 70 | | 30 | |
| 2016 | 75 | | 25 | |
